# The Influence of Hydrated Lime and Cellulose Ether Admixture on Water Retention, Rheology and Application Properties of Cement Plastering Mortars

**DOI:** 10.3390/ma14195487

**Published:** 2021-09-23

**Authors:** Edyta Spychał, Ryszard Dachowski

**Affiliations:** Faculty of Civil Engineering and Architecture, Kielce University of Technology, 25-314 Kielce, Poland; tobrd@tu.kielce.pl

**Keywords:** rheological properties, application properties, water retention, cement, hydrated lime, plaster, cellulose ether, viscosity

## Abstract

In this article, the effect of hydrated lime and cellulose ether on the water retention, rheology, and application properties of plasters was studied. For mortars, the consistency, bulk density, and water retention were tested. Rheological measurements of pastes included yield stress and plastic viscosity. In addition to standard tests of mortars and examining the rheological properties of the pastes, a proprietary method for testing the application properties was proposed. The obtained research results made it possible to evaluate the performance of the tested plasters. An attempt was also made to correlate the rheological properties of pastes (plastic viscosity) to the water retention value. The influence of hydrated lime and cellulose ether on selected properties of pastes and plasters was also presented using the statistical Box–Behnken method. The subjective rating of an expert plasterer confirmed the necessity of the modification of plastering mortars with hydrated lime and cellulose ether. As shown, modification of cement plastering mortar with hydrated lime and cellulose ether at the same time allows obtaining a material with favorable technical and technological properties, especially mortars applied by machine.

## 1. Introduction

The development of new technologies and construction chemicals has contributed to the popularization of dry mortars. The traditionally used cement, cement–lime, and lime plasters are being replaced by materials modified with chemical admixtures and additives. Currently produced mortars consist of several or even a dozen or so raw materials, which are selected so that the final product is characterized by optimal, previously assumed properties. Proper selection of the type and quantity of ingredients in mortars determines the fulfillment of technical and technological requirements. Due to many types and modifications of chemical admixtures, various types of masonry elements, and various substrates, this issue requires a lot of knowledge and practice. A wrong decision during the design of the mortar can lead to failure to meet standard requirements, loss of adhesion, difficulties in plaster application, and the appearance of shrinkage cracks on the material [1,2,3].

One of the most commonly used polymers in dry-mixed mortars is represented by cellulose ethers. They belong to the group of viscosity-modifying admixtures [4,5,6,7], which have been used as ingredients among others of masonry mortars, plasters, and ceramic tile adhesives [2,8,9,10,11,12,13]. Cellulose ethers are important admixtures in dry-mixed mortars because of their thickening effect and water retention ability [14,15,16]. Thanks to this admixture, mortars are easy to prepare, comfortable to use, and easy to handle [2,9].

The water retention and rheological properties of cement or lime mortars have been the subject of many studies. Patural et al. [17] found that the molecular weight is crucial to control water retention and consistency in cement mortars. It was noted that, as molecular weight increased, the yield stress diminished, the consistency increased, and the water retention improved. Betioli et al. [18] studied the effect of cellulose (HEMC) ether on the rheology of pastes and cement hydration. The correlation between the results from rheology and isothermal calorimetry was investigated. According to this study, the rheology of cement pastes changes during the induction period, when the reactivity of the system is low. The rheologic changes are due to the agglomeration of particles. In the first hours of hydration, cellulose ether increased the critical strain of the cement pastes measured using the strain sweep test when compared to the plain cement paste, probably by acting as a steric barrier, in addition to the retardation effect, according to the authors. A higher admixture content results in a higher and longer steric effect because the HEMC molecules adsorbed onto the cement particles and on C–S–H enhance the suspension stability due to steric repulsion. In [4], the effect of cellulose ether and guar gum on aerial lime-based mortars was explored. Standard and rheological investigations were supplemented with mercury intrusion porosimetry. The amount of mixing water was the greatest when cellulose ether was added, and this additive did not increase the water-retention capacity of the fresh mixtures. As assessed by the authors, high water absorption through capillarity, high permeability, and a long delay in setting time resulted in an undesirable general performance of this admixture in aerial lime-based mortars. Hydraulic lime-based mortars modified with cellulose ether were studied by the authors of [5,19,20]. In [5], the behavior of mortars with four different cellulose ethers was assessed. The results revealed an elevated air content and water retention in mortars with an increasing dose of polymer admixture, resulting in the decreased density of mortars in plastic state. The mechanical properties of modified mortars surpassed the reference ones at the age of 180 days despite the fact that these materials showed higher open porosity and water absorption. The enhanced porosity of mortars resulted in an improvement of their frost resistance and faster carbonation. Summarizing the research conducted, the authors stated that the effect of cellulose ether on hydraulic lime mortars is very similar to that on cement mortars, but it is different to that on air lime-based mortars.

In addition to the consistency, water retention, and rheological properties, the influence of cellulose ethers on cement hydration has also been assessed [6,21,22,23]. On the basis of the experimental results, it can be concluded that the addition of cellulose ether has a significant influence on the early hydration of cement, and this effect practically disappears after longer period. Cellulose ethers lead to a gradual slowing down of the C_3_A hydration depending on admixture chemistry. According to observations, substitution groups (nature and content) seem to be more important controlling factors for C_3_A hydration than molecular mass. The amount of the polymer admixture used is most important in the process of setting pastes and mortars. A much-reduced impact is visible when taking into account the viscosity of the admixture or the type of binder used.

The research on cement hydration was supplemented in [23] with a study of the microstructure observations under SEM (after 24 h of hydration). The microstructure of cement paste with cellulose ether had a high content of gypsum crystals. As shown by the EDS analyses of C–S–H in the paste with cellulose ether, this phase had a very low content of aluminum and sulfur. This confirmed the absence of monosulfate in this sample, as the content of aluminum and sulfur in the classical C–S–H phase increases when it forms a nano-mixture with monosulfate. As the author proved, the cellulose ether admixture inhibited the reaction of the C_3_A phase with sulfate ions, which would have led to the formation of ettringite and monosulphate. In [24], the morphology of the hydrate crystals and the microstructure of the hardened cement matrix modified with polyvinyl alcohol–acetate and two cellulose ethers were studied. Polymer modification improved the cohesion of the bulk cement paste, while fewer microcracks were observed for the polymer-modified mortars.

Another analyzed issue in the literature is the influence of cellulose ethers on the shrinkage of mortars [25,26]. Messan et al. [25] presented a new testing method for investigating early-age shrinkage of cement-based material modified with glass fiber, cellulose ether, and redispersible powder. Results of this research showed the importance of the rheology of fresh mortar with regard to early-age shrinkage development. The authors proved that the admixture of cellulose ether decreases the early-age evaporation rate of cement-based materials. This has also been observed in the literature [26]. The use of a viscosity-modifying admixture showed a beneficial effect on mortar shrinkage. According to the authors, the action of cellulose ether reduces the surface tension of the pore solution, thus reducing capillary forces. Long-term water-binding capacity and high-level mortar humidity also contribute to reducing shrinkage deformation.

The nature and the scope of cellulose ether activities are not fully recognized and understood, especially in the case of cement–lime mortars. In addition, there is no information in the literature on how cellulose ether affects their application properties, which is an important issue in the case of plasters. Scientific research in the literature has focused on the assessment of the effect of this admixture on cement [1,9,22,23,27,28], lime [4,5,7,20], and gypsum [29] pastes and mortars. The research carried out by various authors has mainly covered the subject of the influence of this polymer admixture on the standard parameters, consistency, water retention, and rheological properties, among others.

The test results and their analysis presented in this article focus on the assessment of plasters using proprietary methods supplemented with standard methods of pastes and mortars. Importantly, the tests carried out can be applied to both cement-based mortars and cement–lime mortars.

## 2. Materials and Methods

### 2.1. Materials

A commercial Portland cement CEM I 42.5 R (Cemex, Chełm, Poland) [30], deeply separated hydrated lime (Alpol, ZSChiM “PIOTROWICE”, Sitkówka, Poland) [31], two fractions of quartz sand 0.1–0.5 mm and 0.2–0.8 mm (Grudzeń Las, Grudzeń Las, Poland) [32], a polymer admixture of different viscosity and amount (WALOCEL, The Dow Chemical Company, Midland, MI, USA) [33], and tap water were used. Among the wide variety of cellulose ethers, the following were chosen in this research: hydroxypropyl methylcellulose (HPMC) with viscosity 3000 mPa·s and hydroxyethyl methylcellulose (HEMC) with viscosity 25,000 mPa·s and 45,000 mPa·s (for measurements using a Brookfield rheometer at 20 °C). These polymer admixtures had the form of a very fine white powder, with a grain size of less than 0.063 mm and a low degree of chemical modification. The chemical composition and physical properties of cement are given in Table 1. The chemical composition of hydrated lime is shown in Table 2. 

Silicate blocks of N12 type with dimensions 0.25 m × 0.12 m × 0.22 m (H+H Company, Warsaw, Poland) [34] were used to test the application properties of plasters (for bricklaying the walls). The basic properties of these masonry elements are presented in Table 3.

### 2.2. Mix Proportions of Pastes and Mortars

The Box–Behnken experiment plan was used to analyze the plasters [35,36]. Pastes and mortars were tested by taking into account the selected experimental plan. The analysis of the test results was performed using the STATISTICA computer program (StatSoft Company, Cracow, Poland) [37]. A solution was selected, as a result of which it was possible to conduct research for a three-factor, three-level model. A matrix with values of the coded factors is presented in Table 4, while a matrix with selected types of mortars and their composition is presented in Table 5. According to the selected experiment plan, it was necessary to carry out 15 elementary experiments each time—13 different experiments (13 mixtures of different compositions) and two repetitions (for the mixture corresponding to the central point of the Box–Behnken plan). Additionally, a base cement mortar was prepared and tested, which contained neither hydrated lime nor cellulose ether (paste and mortar marked with the symbol C0). The composition of the pastes was similar to the composition of the mortars, excluding fine aggregate.

Factors that were kept constant throughout this study were as follows:

- Total amount of binder in the mortar (96 g of binder per 1000 g of dry ingredients);
- Ratio of binder to fine aggregate (1:10);
- Consistency measured using a flow table (165 mm).

On the basis of the experiments carried out in accordance with the Box–Behnken model, it was possible to determine the quadratic formula (Equation (1)) for the tested property in each case, which was used to estimate the response area [36,38]. In Equation (1), Y_i_ is the dependent variable or output variable (tested parameter of pastes/mortars), b_0_–b_33_ are factors, and X_1_, X_2_, and X_3_ are dependent variables (input factors). For the tested properties of pastes and mortars, the value of the standard deviation was determined for the central point of the Box–Behnken experiment plan, i.e., sample numbers 13, 14, 15 (mortar abbreviated as C25-18.82MV).

Y_i_ = b_0_ + b_1_X_1_ + b_2_X_2_ + b_3_X_3_ + b_11_X_1_^2^ + b_22_X_2_^2^ + b_33_X_3_^2^ + b_12_X_1_X_2_ + B_12_X_1_X_3_ + b_23_X_2_X_3_. (1)

### 2.3. Methods

All pastes and mortars were prepared in an air-conditional laboratory at a temperature of 20 ± 2 °C and a relative humidity of 65% ± 5%. Binders, fine aggregate, and water were weighed with an accuracy of 0.1 g, and the chemical admixture was weighed with an accuracy of 0.0001 g. The mixing method was the same for all samples: 90 s of mechanical stirring at 45 rpm + 30 s pause + 90 s of mechanical stirring at 57 rpm.

Mortar consistency was assessed using two methods: a flow table according to the PN-EN 1015-3:2000 standard [39] and the fall cone method according to the PN-B-04500:1985 standard [40]. The consistency was measured using the method in [39] as the average of two measurements of the mortar flow on the flow table. The consistency measured using the method in [40] was defined as the depth of immersion of the cone in the tested mortar.

The bulk density of fresh mortars was calculated according to PN-EN 1015-6:2000 + A1:2007 standard [41].

The water retention value (WRV) was determined according to the procedure described in [42]. This parameter denotes the percentage water content remaining in the tested mortar after short-term contact with the filter paper. Each mortar was tested three times, determining its value after 10, 30, and 60 min, e.g., WRV10, WRV30, and WRV60. This nonstandard test allowed evaluating the behavior of the mortar in contact with a material (masonry element) with varied adsorption capacity.

Rheological measurements were carried out using the Viskomat NT rheometer (Schleibinger Testing Systems, Buchbach, Germany). The rheological behavior of pastes is described by the Bingham model [43,44,45], using g (N·mm) and h (N·mm·s) as parameters describing yield stress and plastic viscosity. These properties of the pastes were determined on the basis of the flow curves for decreasing shear rates, after 10 and 60 min. The temperature of pastes between individual measurements was constant (20 °C), set by an automatic thermostatic system. Each sample was well protected against water evaporation by being placed in a sealed container for the periods between studies.

**Figure 1:**
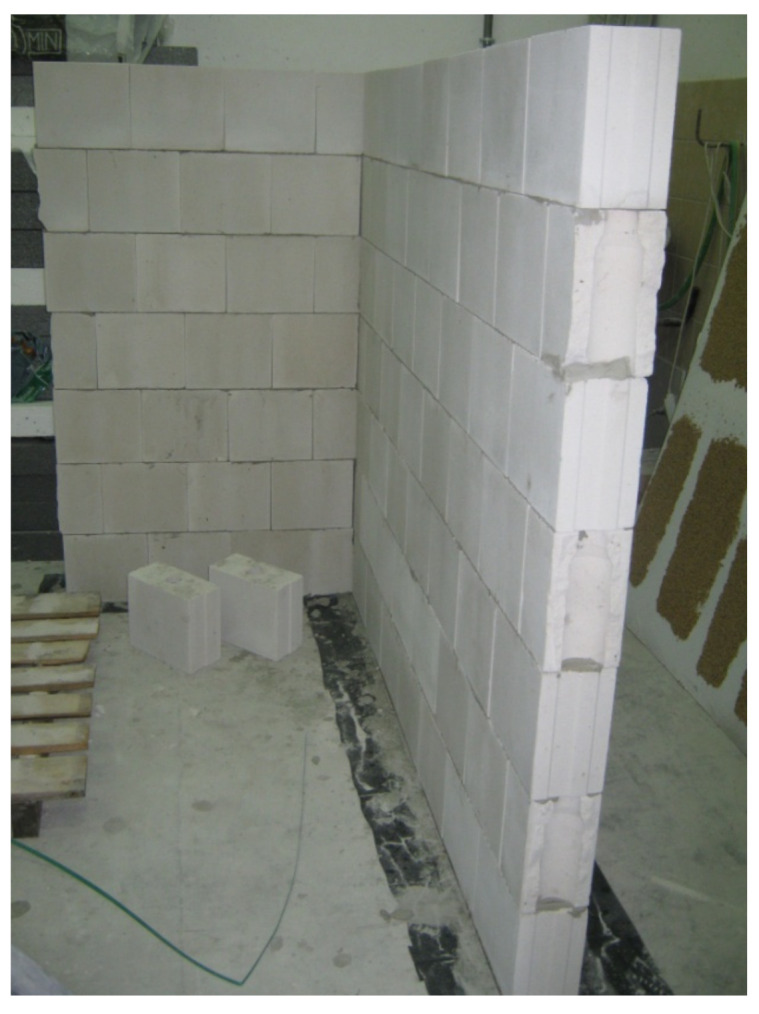
View of the wall before plastering.

The application properties of mortars were estimated on the basis of a subjective rating by an expert plasterer (Edyta Spychał’s original method, which was used in her Ph.D. thesis) [46]. The first stage was to build a wall of silicate blocks (the masonry elements were seasoned for a period of 30 days in the research conditions). Then, the wall was primed. An image of the laid brick walls is shown in Figure 1, one with dimensions of 87.5 cm width and 154 cm height, and the other with dimensions of 137.5 cm width and 154 cm height. 

The assessment of mortars was carried out in two stages. On the first day, a specialist plastered the walls and evaluated the fresh materials in terms of consistency, viscosity, the ease of plastering, adhesion to the substrate, and potential difficulties during plastering. On the second day, the ease of surfacing (smoothing, surface correction) was determined.

The analysis of application properties consisted of the following elements:

1. Evaluation of application properties:
  - On a 0–10 point scale (10—material with the best application properties);
  - Based on a detailed descriptive assessment;
  - Based on the possible method of application: manual and/or machine-assisted).
2. Assessment of the ease and quality of surfacing:
  - On a 0–10 point scale (10—material with the best properties);
  - Based on a detailed descriptive assessment.

This article is a part of a wider analysis in which the influence of cellulose ether and hydrated lime on the selected properties of plasters was evaluated [46]. Some research in this direction was previously introduced in [2,6,10].

## 3. Results

### 3.1. Consistency and Bulk Density Measurements

In Table 6, the results of the amount of water in mortars, the consistency, and the bulk density for all samples are presented.

**Figure 2:**
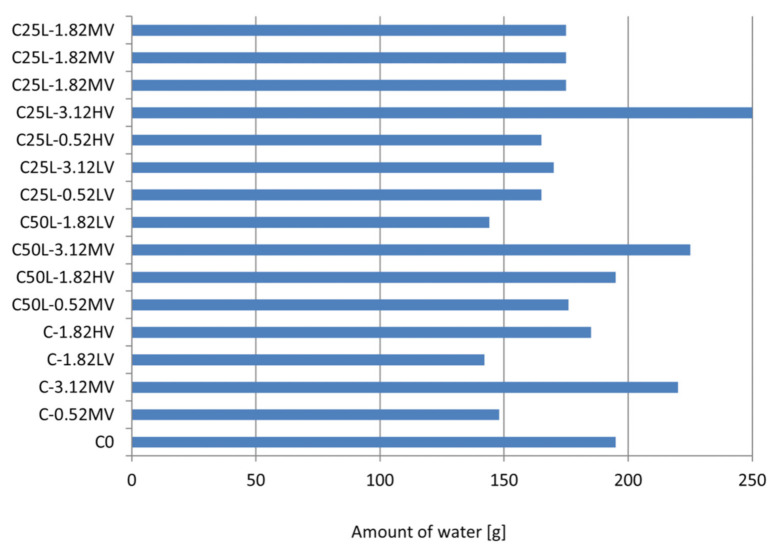
Amount of water in mortars.

The consistency of the mortars measured using a flow table was selected experimentally, in accordance with the assumptions of the research, i.e., at a constant level of 165 mm. According to the values given in Table 6 and Figure 2, it can be seen that the amount of water needed to obtain the required consistency was highly variable and ranged from 142 g to 250 g. The amount of water needed for the reference mortar (C0) was 195 g. For the three mortars C-3.12MV, C50L-3.12MV, and C25L-3.12 HV, to obtain the required consistency, at least 220 g of water was required. This value was mainly due to the viscosity of cellulose ether used, which, in these cases, was 25,000 mPa·s and 45,000 mPa·s, as well as the maximum added amount of cellulose ether, which, in these cases, was 3.12%. Hence the conclusion, than when selecting the required amount of water, the amount and viscosity of this admixture first of all should be taken into account.

Obtaining consistency at the same level for cement and cement–lime mortars modified with cellulose ether was associated with an increase in the amount of water when replacing a part of the cement binder with lime. This was due to the smaller grain size of lime compared to the grain size of cement, thus leading to its higher water demand (C-0.52MV and C50L-0.52MV mortars).

The use of cellulose ether in mortars is associated with a greater demand for water in order to obtain a consistency at a similar level compared to unmodified mortar. An increase in the admixture content is usually associated with an increase in the water demand of the mortar [3,12]. This research shows that this relationship was not always true, as observed for the examples of C-0.52MV, C-1.82LV, C50L-1.82LV, C25L-0.52LV, and C25L-0.52HV materials. These plasters required less water to achieve the assumed consistency, compared to the reference mortar C0. The reasons for this phenomenon are due to the properties of cellulose ether. When the viscosity of the liquid phase surrounding the grains of the binder and fine aggregate is too low, the dissolved polymer acts as a “lubricant”, facilitating the movements of the mortar grains [29,47]. In this case, the action of the cellulose ether is similar to that of a plasticizing or fluidizing admixture.

**Figure 3:**
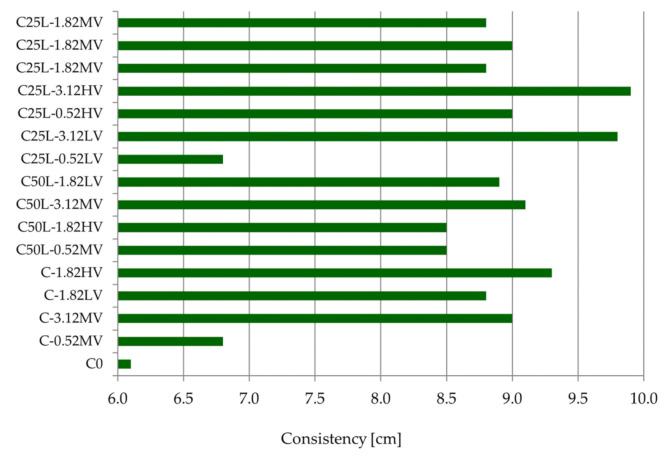
Consistency results of mortars according to PN-B-04500:1985.

Taking into account the test results from Table 6 and Figure 3, it can be concluded that the consistency of the mortars varied, ranging from 6.1 cm (C0) to 9.9 cm (C C25L-3.12HV). The standard deviation of the consistency test results [40] was equal to 0.094 cm. All tested mortars achieved a consistency similar to that of typical plasters used in practice [42,48]. The consistency of plastering mortars intended for manual application should be 6–9 cm, whereas the equivalent value for mechanical application should be 8–11 cm. The results obtained in this research fall within the range of consistency given in the literature [48].

**Figure 4:**
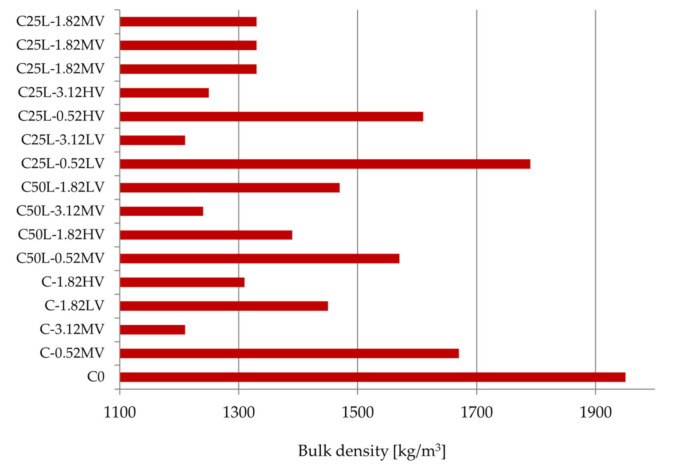
Results of bulk density of fresh mortars.

Table 6 and Figure 4 present the range of bulk density values of fresh mortars. The standard deviation of the bulk density test results was equal to 0.000 kg/m^3^. The lowest bulk density was achieved using the cement mortar modified with cellulose ether in the amount of 3.12% with a viscosity of 25,000 mPa·s (1210 kg/m^3^—C3.12-MV), while the highest value was achieved by the unmodified mortar (C0)—1950 kg/m^3^. This parameter can be used to indirectly judge the performance of the plasters (raw material efficiency). The material with the lowest bulk density is characterized by the best performance. Mortar efficiency is important not only from an economic but also from a technological point of view. All modified mortars had a lower bulk density than the base mortar, which reduced the consumption of materials. Thus, cellulose ether and hydrated lime had a positive effect on increasing the efficiency of the tested mortars, as well as reduced the bulk density. This result is in agreement (for cement mortar) with the literature [3,13].

### 3.2. Water Retention of Mortars

**Figure 5:**
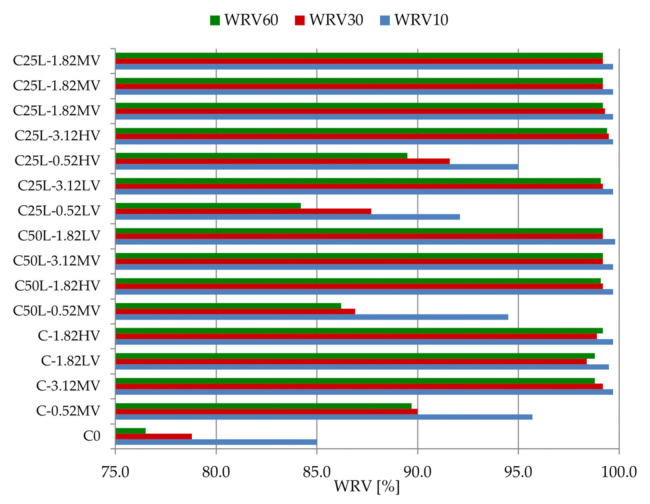
Change in water retention of tested mortars over time (WRV parameter).

Table 7 and Figure 5 present the results of the water retention value (WRV) after 10, 30, and 60 min. The standard deviation of the WRV test results was equal to 0.000% for WRV10 and WRV60 and equal to 0.047% for WRV30.

Comparing the WRV parameter of the mortars, it can be seen that the reference mortar C0 had an initial value approximately 10–15% lower than that of the other mortars. After 15 min, is the water retention was 85.0%; however, after 60 min, it was 76.5%, which is a disadvantage, especially when the plaster is applied on a substrate with high absorbency or when plastering take place in conditions of changing temperature and humidity. In the case of materials C-0.52MV, C50L-0.52MV, C25L-0.52MV, and C25L-0.52MV WRV10, the water retention was greater than 90%, decreasing after 30 and 60 min due to the lowest cellulose ether content of 0.52% in each case. This proves the significant influence of the amount of admixture in terms of water retention in the mortar and its action over time. For the remaining materials, the water retention value remained at a level close to 100% throughout the entire study. Comparing C50L-1.82HV and C50L-1.82LV mortars with the same proportions of binder and amount of admixture, there was no visible influence of the polymer viscosity. The partial change from cement to lime binder was not as noticeable and important as the change in the amount of cellulose ether. According to Brumaud et al. [15], mortars can be classified as follows: C0 mortar would be a material with low water retention (WRV < 86%), C25L-0.52LV mortar would be characterized by medium water retention (86% < WRV < 94%), and the remining samples would be considered as having high water retention (WRV > 94%).

**Figure 6:**
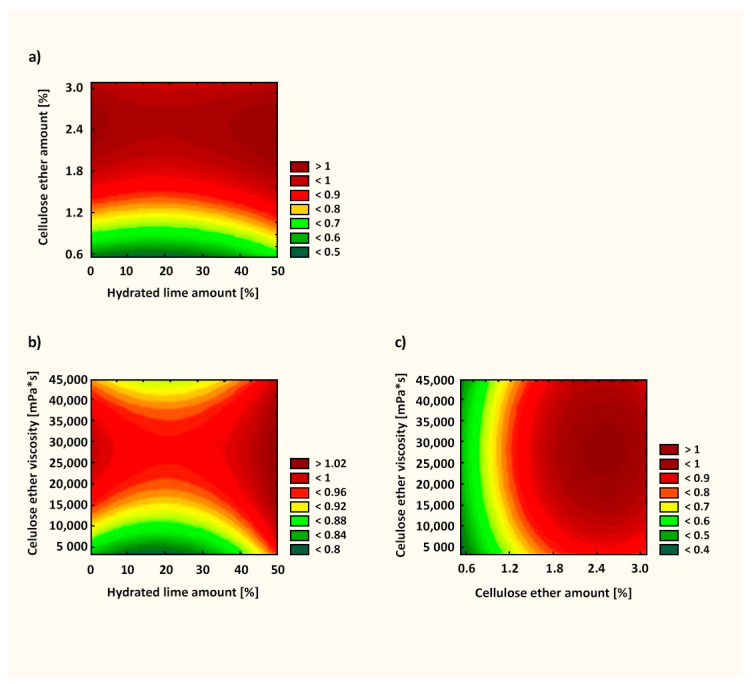
Utility function for the WRV10 test of mortars: (**a**) correlation between cellulose ether amount and hydrated lime amount; (**b**) correlation between cellulose ether viscosity and hydrated lime amount; (**c**) correlation between cellulose ether viscosity and cellulose ether amount.

When assessing the test results of the water retention value in mortars after 10 min of testing, similar relationships were obtained in comparison with the literature data. The presence of cellulose ether in cement or lime mortars increases water retention [7,16,49]. Information about the effective action of the admixture in cement–lime mortars, which is not available in the literature, was confirmed in this article. In the case of mortars with a cement–lime binder, a strong influence of the polymer in terms of water retention was visible throughout the entire measurement. Figure 6 presents the response surface of the influence of X_1_, X_2_, and X_3_ factors on WRV10 (Y_1_). The fit factor of the *R*^2^ model in this case was 0.962, which denotes that this model explained 96.2% of changes in the response value. As can be seen from Equations (2)–(4), the most important factor influencing WRV10 was the amount of cellulose ether (X_2_ factor). For this factor, the significance level was *p* < 0.05, suggesting that the X_2_ factor had a statistically significant influence on the examined feature. Statistically, the amount of hydrated lime and cellulose ether viscosity had a lesser effect on the parameter tested. As the amount of admixture in the mortars increased, the WRV10 increased. Thus, modifying plasters with cellulose ether is a favorable solution from the point of view of water retention in the mortar. The utility level shown in the range from 0 to 1 in Figure 6 determines the influence of the defined factors on the tested parameter, suggesting the optimal arrangement of the factors X_1_, X_2_, and X_3_. Adding as little as 1.2% cellulose ether enabled obtaining a high WRV10 (Figure 6).

Y_1_ = 91.519 − 0.06X_1_ + 0.0016X_1_^2^ + 6.002X_2_ − 1.213X_2_^2^, (2)

Y_1_ = 91.519 − 0.06X_1_ + 0.001X_1_^2^ + 0.0001X_3_, (3)

Y_1_ = 91.519 + 6.002X_2_ − 1.213X_2_^2^ + 0.0001X_3_. (4)

### 3.3. Rheological Properties of Pastes

**Figure 7:**
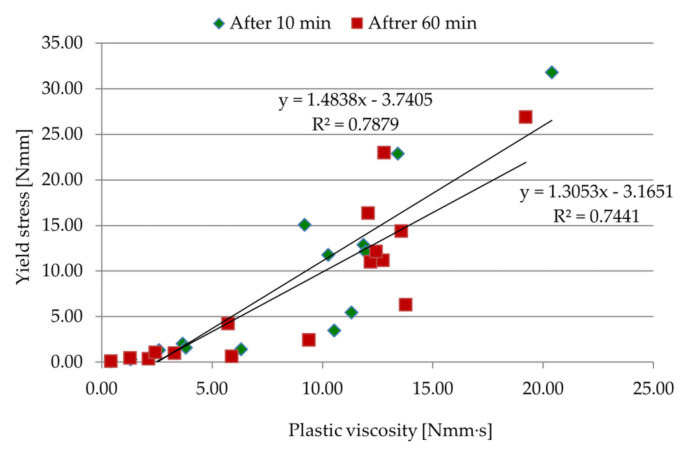
Correlation between yield stress and plastic viscosity of pastes.

**Figure 8:**
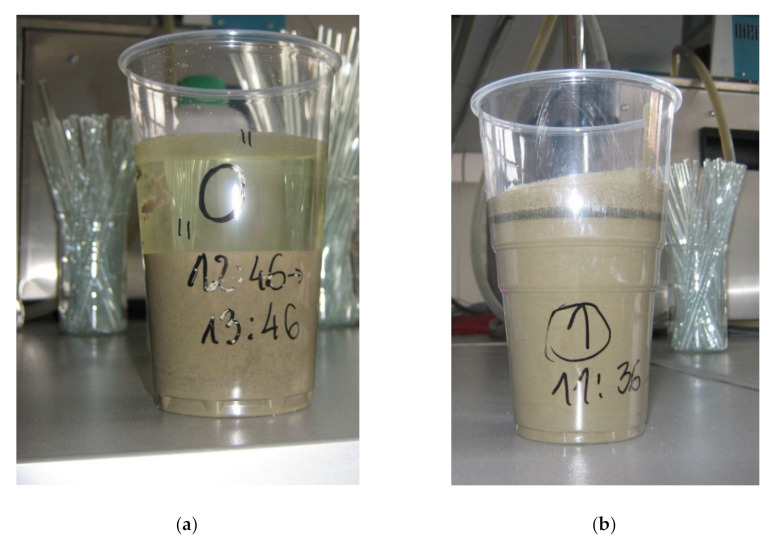
Photos showing sedimentation of pastes: (**a**) paste C0; (**b**) paste C-0.52MV.

Table 8 and Figure 7 present the results of the rheological properties of pastes after 10 and 60 min. The standard deviation of the yield stress test results was equal to 0.354 N·mm for the measurements after 10 min and 0.514 N·mm for the measurements after 60 min, whereas the standard deviation of the plastic viscosity test results was equal to 0.052 N·mm·s for measurements after 10 min and 0.233 N·mm·s for the measurements after 60 min. When analyzing the above test results, it can be seen that the yield stress and plastic viscosity of the reference paste were the smallest. With an increase in the amount and viscosity of cellulose ether, both parameters increased after 10 and 60 min. Paste C50L-3.12MV with 50% hydrated lime content, cellulose ether content 3.12%, and viscosity 25,000 mPa·s had the biggest yield stress and plastic viscosity. The test results obtained for C50L-1.82HV, C50L-3.12MV, C25L-3.12HV, and C25L-1.82MV pastes, which translated into large values of rheological parameters, were associated with a higher percentage of cellulose ether and a high viscosity, in combination with the replacement of a part of the cement binder with hydrated lime binder. Information in addition to the measurement results of rheological parameters was also provided by the observations of the paste samples during the research. It is known that hydrated lime and cellulose ether affect the workability and plasticity of the material, as well as its cohesion, which was confirmed during the rheological tests. The appearance of C0 and C-0.52MV pastes after 60 min differed from that of the other samples, as shown in Figure 8a,b. In particular, visible differences were observed for the C0 paste. In the case of this sample (C0 paste), a clear separation of water from the rest of the ingredients could be noticed. The separation of water in the C-0.52MV paste was also visible, but to a smaller extent. Both samples (C0 and C-0.52MV) were characterized by the sedimentation of ingredients and a lack of homogeneity through the mass, confirming the successful modification of the pastes and mortars. Despite the fact that the C-0.52MV paste was modified with an admixture, the amount of cellulose ether seemed to be insufficient to prevent segregation of the ingredients of the paste. Comparing samples with the same amount and viscosity of cellulose ether, but differing in the type of binder (C-0.52MV and C50L-0.52MV), a beneficial effect of hydrated lime was observed. In sample C50L-0.52MV, there was no visible sedimentation of the components, while the leaven was coherent and homogeneous throughout the mass.

A correlation between plastic viscosity and yield stress was established, as shown in Figure 7. The trend showed an increase in yield stress with the increase in plastic viscosity, which is consistent with the action of hydrated lime and cellulose ether. According to [50], there is a high correlation and significant interdependence of the examined parameters.

**Figure 9:**
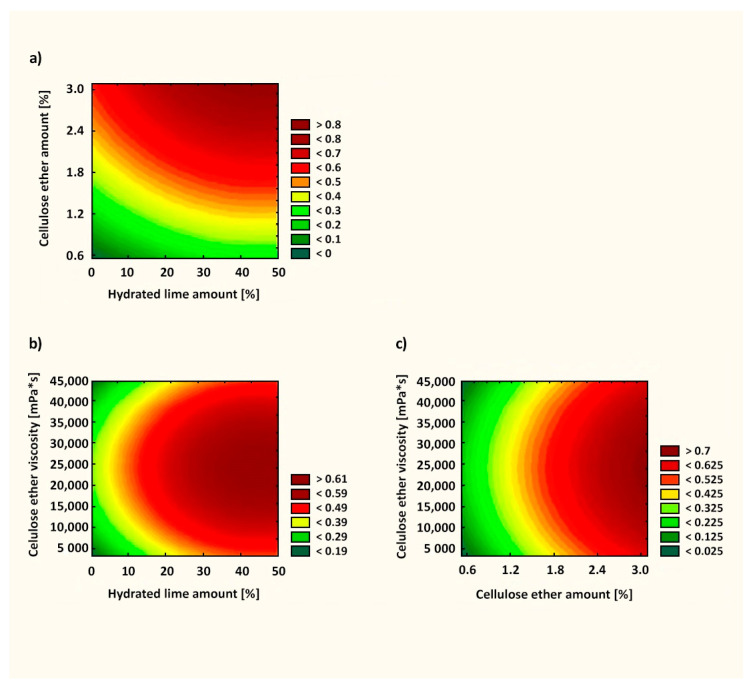
Utility function for the plastic viscosity after 10 min of pastes: (**a**) correlation between cellulose ether amount and hydrated lime amount; (**b**) correlation between cellulose ether viscosity and hydrated lime amount; (**c**) correlation between cellulose ether viscosity and cellulose ether amount.

Figure 9 presents the response surface of the influence of X_1_, X_2_, and X_3_ factors on the plastic viscosity after 10 min (Y_2_). The fit factor of the *R*^2^ model in this case was 0.902. As can be seen from Equations (5)–(7), the most important factor influencing the plastic viscosity was the amount of cellulose ether (X_2_ factor). Statistically, the amount of hydrated lime and cellulose ether viscosity had a lesser effect on the parameter tested. As the amount of admixture in the mortars increased, the plastic viscosity increased (similar to the statistical evaluation of the WRV).

Y_2_ = − 7.031 + 0.226X_1_ − 0.002X_1_^2^ + 7.641X_2_ − 0.898X_2_^2^, (5)

Y_2_ = − 7.031 + 0.226X_1_ − 0.002X_1_^2^ + 0.0003X_3_, (6)

Y_2_ = − 7.031 + 7.641X_2_ − 0.898X_2_^2^ + 0.0003X_3_. (7)

### 3.4. Application Properties of Mortars

Table 9 shows the scoring of the quality of plastering mortars, the results of the assessment of the performance of the tested materials, and the methods of application of the plasters. The C-0.52MV, C-1.82HV, C50L-1.82HV, and C25L-1.82MV mortars received the highest score, followed by the C-1.82LV, C50L-0.52MV, and C50L-1.82LV mortars. The remaining plasters were of very poor quality. According to a specialist plasterer, mortars with the highest scores were those can be used as commercial products without any additional changes in their composition, whereby some of the tested mortars could be applied manually and others could be applied by machine. Mortar C50L-1.82HV was deemed a universal material, which could be applied manually or by machine. The methods of application were determined on the basis of the rheological properties. Mortars fit for a manual method of application had low plastic viscosity, whereas those fit for machine application had high viscosity. High plastic viscosity facilitates the application of the mortar, whereby the material clings to the float and does not run off the wall. Hydrated lime also improved the flexibility of the plasters and the ease of processing. Hydrated lime and cellulose ether were essential in the case of machine-applied plasters due to the method specificity. In addition, hydrated lime facilitated plaster processing, and cellulose ether enabled longer water retention in the mortar.

**Figure 10:**
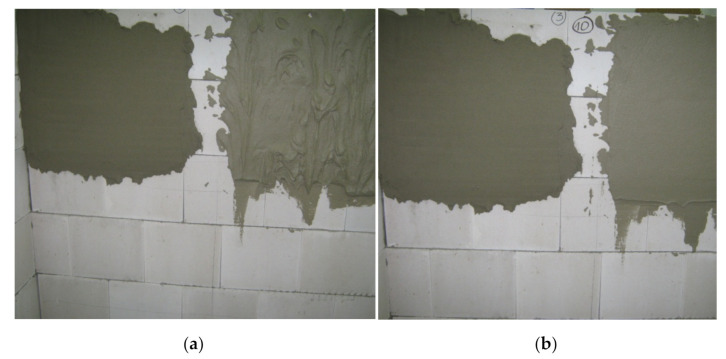
Images of walls (**a**) during application, and (**b**) during processing.

Figure 10 presents images of the walls during application (first-day assessment of application properties) and processing (second-day assessment). 

Due to the fact that the point assessment did not fully reflect the properties of each mortar, the results on the point scale were supplemented with a descriptive assessment. Table 10 shows a description of the application properties of the selected mortars. Materials were also visually assessed after 28 days of setting and hardening processes. Mortars containing hydrated lime in their composition had a much lighter color, which is advantageous from a technological and economic point of view. This allows a reduction in paint consumption for coating.

**Figure 11:**
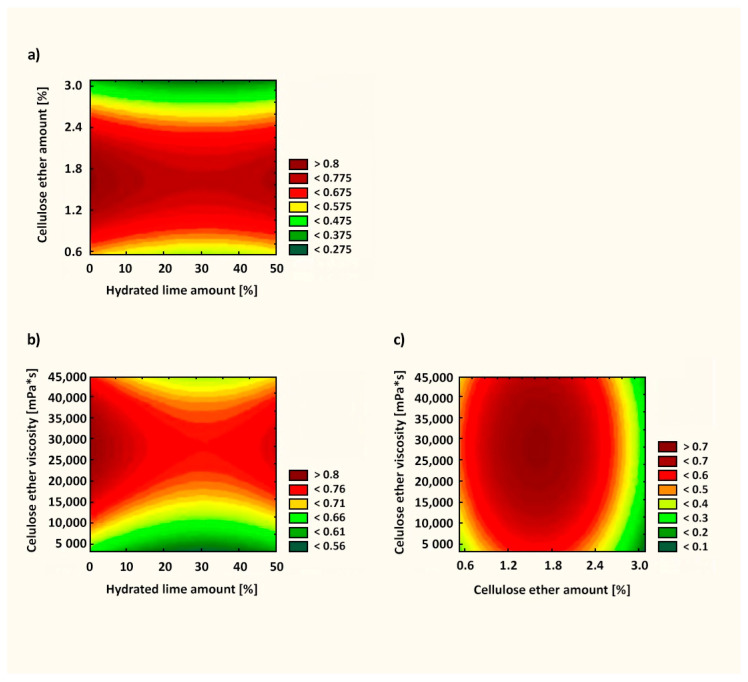
Utility function for subjective rating of mortars by a specialist: (**a**) correlation between cellulose ether amount and hydrated lime amount; (**b**) correlation between cellulose ether viscosity and hydrated lime amount; (**c**) correlation between cellulose ether viscosity and cellulose ether amount.

Figure 11 presents the response surface of the influence of X_1_, X_2_, and X_3_ factors on the subjective rating by a specialist (Y_3_). The fit factor of the *R*^2^ model in this case was 0.749. Statistically, as can be seen from Equations (8)–(10), the most important factor was the amount of cellulose ether (X_2_ factor). Adding as little as 1.82% cellulose ether led to favorable application properties (Figure 11).

Y_3_ = 0.685 − 0.11X_1_ − 0.0018X_1_^2^ + 13.932X_2_ − 4.29X_2_^2^, (8)

Y_3_ = 0.685 − 0.11X_1_ − 0.0018X_1_^2^ + 0.0003X_3_, (9)

Y_3_ = 0.685 + 13.932X_2_ − 4.29X_2_^2^ + 0.0003X_3_. (10)

### 3.5. Correlation between Rheological Properties of Pastes and Chosen Properties of Mortars

**Figure 12:**
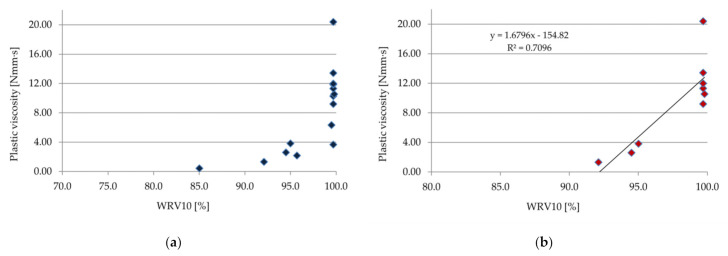
Correlation between plastic viscosity and WRV10 (**a**) for all samples, and (**b**) only for samples with cement–lime binder.

**Figure 13:**
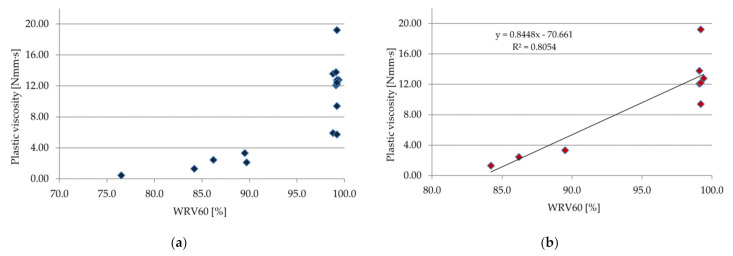
Correlation between plastic viscosity and WRV60 (**a**) for all samples, and (**b**) only for samples with cement–lime binder.

A correlation between the rheological properties (plastic viscosity) and WRV was established, as shown in Figure 12 (correlation experimental results after 10 min) and in Figure 13 (correlation experimental results after 60 min). Comparing the individual test results, one can notice a high correlation and interdependence of the plastic viscosity and WRV, especially for materials based on a cement–lime binder (Figure 12b and Figure 13b). The trend showed an increase in WRV with the increase in plastic viscosity. For pastes with a plastic viscosity over 8 N·mm·s, WRV10 and WRV60 were all within the limits. Similar correlations between the rheological properties and water retention value were obtained in the case of cement and lime pastes and mortars in [49,51].

## 4. Conclusions

On the basis of the results presented in this a article, the following conclusions can be derived:

- Modification of cement plastering mortar with both hydrated lime and cellulose ether allows obtaining a material with favorable technical and technological properties, especially for mechanical application.
- Correctly selected proportions of hydrated lime and polymer admixture with a specific viscosity ensure appropriate application properties: high efficiency, workability, and ease of plastering.
- An increase in the plastic viscosity of pastes influences the increase in WRV of the mortars.
- An increase in the amount and viscosity of cellulose ether increases the plastic viscosity and the yield stress.
- The subjective assessment of a specialist plasterer and the rheological parameters are valuable sources of information for determining the application properties of mortars. Indirectly, the results of these studies allow determining the method of mortar application.
- Providing the right consistency or obtaining a high WRV does not guarantee a high-quality material in terms of meeting the standard requirements and workability.
- The influence of cellulose ether on the tested properties of pastes and mortars (WRV, as well as rheological and application properties) is statistically more significant than the influence of hydrated lime.
- After concluding the research presented in the article, the authors propose that, related to the water retention mechanisms, the rheological properties of the mortar are inluential properties, but they should not be the main determinant in assessing the application properties of plasters or in selecting the method of mortar application.

## Figures and Tables

**Table 1:** Chemical composition and physical properties of cement.

Chemical Composition (wt.%)		Physical Properties	
SiO_2_	20.22	Water requirement of normal consistency (%)	28.8
Al_2_O_3_	4.43	Initial setting (min)	173
Fe_2_O_3_	3.65	Final setting (min)	237
CaO	64.06	Specific surface area (m^2^/kg)	387.9
Na_2_O	0.29	2 day compressive strength (MPa)	28.9
MgO	1.24	28 day compressive strength (MPa)	59.5
SO_3_	3.31	2 day flexural strength (MPa)	5.1
Cl	0.093	28 day flexural strength (MPa)	8.4
K_2_O	0.54	Loss on ignition (%)	3.81
Free CaO	1.83		

**Table 2:** Chemical composition of hydrated lime.

Chemical Composition (wt.%)	
CaO + MgO	95.17
MgO	0.80
CO_2_	1.86
SO_3_	0.41

**Table 3:** Chosen properties of silicate blocks.

Water absorption (%)	14 ± 2
Compressive strength (MPa)	15.0
Bond strength with PN-EN 998-2 standard, GPM ^1^ (MPa)	0.15
Bond strength with PN-EN 998-2 standard, TLM ^2^ (MPa)	0.30
Heat conductivity (W/m·K)	0.46
Average block weight (kg)	9.5

^1^ For general-purpose mortars and light mortars; ^2^ for thin pointing mortars.

**Table 4:** Box–Behnken experiment plan.

No.	Experiment Plan			Experiment Plan		
	X_1_	X_2_	X_3_	X_1_ Amount of Hydrated Lime ^1^ %	X_2_ Amount of Cellulose Ether ^2^ %	X_3_ Viscosity of Cellulose Ether mPa·s
1	−1	−1	−1	0	0.52	25,000
2	−1	1	0	0	3.12	25,000
3	−1	0	−1	0	1.82	3000
4	−1	0	1	0	1.82	45,000
5	1	−1	0	50	0.52	25,000
6	1	0	1	50	1.82	45,000
7	1	1	0	50	3.12	25,000
8	1	0	−1	50	1.82	3000
9	0	−1	−1	25	0.52	3000
10	0	1	−1	25	3.12	3000
11	0	−1	1	25	0.52	45,000
12	0	1	1	25	3.12	45,000
13	0	0	0	25	1.82	25,000
14	0	0	0	25	1.82	25,000
15	0	0	0	25	1.82	25,000

^1^ The percentage of hydrated lime given in relation to the total amount of binder in the mortar; ^2^ the percentage of cellulose ether given in relation to the amount of binder in the mortar.

**Table 5:** Mortar and paste compositions defined according to the Box–Behnken model.

Abbreviation of Mortar (Paste)	Amount of Binder (Cement + Lime) (g)	Amount of Sand 0.1–0.5 mm (g)	Amount of Sand 0.2–0.8 mm (g)	Amount of Cellulose Ether (g)
C0 ^1^	96	437	467	-
C-0.52MV	96	437	467	0.50
C-3.12MV	96	437	467	3.00
C-1.82LV	96	437	467	1.75
C-1.82HV	96	437	467	1.75
C50L-0.52MV	48 + 48	437	467	0.50
C50L-1.82HV	48 + 48	437	467	1.75
C50L-3.12MV	48 + 48	437	467	3.00
C50L-1.82LV	48 + 48	437	467	1.75
C25L-0.52LV	72 + 24	437	467	0.50
C25L-3.12LV	72 + 24	437	467	3.00
C25L-0.52HV	72 + 24	437	467	0.50
C25L-3.12HV	72 + 24	437	467	3.00
C25L-1.82MV	72 + 24	437	467	1.75
C25L-1.82MV	72 + 24	437	467	1.75
C25L-1.82MV	72 + 24	437	467	1.75

^1^ Base mortar (paste) without hydrated lime or cellulose ether.

**Table 6:** Selected properties of fresh mortars.

Symbol of Mortar	Amount of Water (g)	Consistency (cm)	Bulk Density (kg/m^3^)
C0	195	6.1	1950
C-0.52MV	148	6.8	1670
C-3.12MV	220	9.0	1210
C-1.82LV	142	8.8	1450
C-1.82HV	185	9.3	1310
C50L-0.52MV	176	8.5	1570
C50L-1.82HV	195	8.5	1390
C50L-3.12MV	225	9.1	1240
C50L-1.82LV	144	8.9	1470
C25L-0.52LV	165	6.8	1790
C25L-3.12LV	170	9.8	1210
C25L-0.52HV	165	9.0	1610
C25L-3.12HV	250	9.9	1250
C25L-1.82MV	175	8.8	1330
C25L-1.82MV	175	9.0	1330
C25L-1.82MV	175	8.8	1330

**Table 7:** Water retention results.

Symbol of Mortar	WRV10 (%)	WRV30 (%)	WRV60 (%)
C0	85.0	78.8	76.5
C-0.52MV	95.7	90.0	89.7
C-3.12MV	99.7	99.2	98.8
C-1.82LV	99.5	98.4	98.8
C-1.82HV	99.7	98.9	99.2
C50L-0.52MV	94.5	86.9	86.2
C50L-1.82HV	99.7	99.2	99.1
C50L-3.12MV	99.7	99.2	99.2
C50L-1.82LV	99.8	99.2	99.2
C25L-0.52LV	92.1	87.7	84.2
C25L-3.12LV	99.7	99.2	99.1
C25L-0.52HV	95.0	91.6	89.5
C25L-3.12HV	99.7	99.5	99.4
C25L-1.82MV	99.7	99.3	99.2
C25L-1.82MV	99.7	99.2	99.2
C25L-1.82MV	99.7	99.2	99.2

**Table 8:** Rheological properties.

Abbreviation of Paste	Yield Stress after 10 min, g (N·mm)	Yield Stress after 60 min, g (N·mm)	Plastic Viscosity after 10 min, h (N·mm·s)	Plastic Viscosity after 60 min, h (N·mm·s)
C0	0.09	0.08	0.43	0.43
C-0.52MV	0.48	0.35	2.17	2.13
C-3.12MV	11.78	14.38	10.26	13.58
C-1.82LV	1.43	0.62	6.32	5.89
C-1.82HV	2.02	4.22	3.66	5.73
C50L-0.52MV	1.31	1.07	2.60	2.43
C50L-1.82HV	15.06	16.33	9.19	12.07
C50L-3.12MV	31.81	26.89	20.39	19.21
C50L-1.82LV	3.48	2.43	10.54	9.40
C25L-0.52LV	0.32	0.46	1.32	1.30
C25L-3.12LV	5.47	6.29	11.31	13.78
C25L-0.52HV	1.61	0.97	3.82	3.31
C25L-3.12HV	22.86	22.98	13.42	12.79
C25L-1.82MV	12.13	10.97	11.98	12.18
C25L-1.82MV	12.88	11.15	11.86	12.75
C25L-1.82MV	12.13	12.14	11.95	12.43

**Table 9:** Application properties of mortars.

Abbreviation of Mortar	The First Stage of the Assessment	The Second Stage of the Assessment	Manual Method of Application	Machine-Assisted Method of Application
C0	2	0	+	−
C-0.52MV	8.5	8	+	−
C-3.12MV	3	1	+	−
C-1.82LV	3	6	+	−
C-1.82HV	9	8	+	−
C50L-0.52MV	2	6	+	−
C50L-1.82HV	8	8	+	+
C50L-3.12MV	3.5	3.5	−	+
C50L-1.82LV	3.5	8	+	−
C25L-0.52LV	2	6	+	−
C25L-3.12LV	4	1	+	−
C25L-0.52HV	4	1	+	−
C25L-3.12HV	4	1	−	+
C25L-1.82MV	7	8	−	+
C25L-1.82MV	7	8	−	+
C25L-1.82MV	7	8	−	+

**Table 10:** Descriptive assessment of the workability of selected mortars.

Symbol of Mortar	Evaluation of Workability	Evaluation of Processing Quality
C0	Wet sand consistency; low viscosity	Lack of processing capabilities
C-0.52MV	Consistency good for manual coating; low efficiency of plaster; good workability properties	Plaster binds evenly, with good processing time
C-3.12MV	The consistency is very fluid, sticks too much to the float, and runs off the wall after a long time	Plaster is dry on top, wet inside, and characterized by a false bonding, along with difficult processing
C50L-1.82HV	Material with the best application properties and easy application; plaster clings to the float; consistency is very good; plaster is efficient	Plaster does not need water for final treatment

## Data Availability

No new data were created or analyzed in this study. Data sharing is not applicable to this article.
